# Chile: The Challenge of Providing Relevant Information from ILSA Studies for the Improvement of Educational Quality

**DOI:** 10.1007/978-3-030-59031-4_3

**Published:** 2020-11-24

**Authors:** Ema Lagos

**Affiliations:** Mathematics and Statistics, ISEG, University of Lisbon, Lisbon, Portugal; PISA National Coordinator, National Agency for Educational Quality, Morandé 360, Piso 10, Santiago, Chile

## Abstract

Chile has a consolidated culture of evaluation in its educational system because, for more than three decades, first the Ministry of Education and currently the National Agency for Educational Quality have implemented national census tests every year to monitor the established curricula’ learning. International Large-scale Students Assessment (ILSA) studies have substantially contributed to this monitoring since the late 1990s. Both, the definition of the disciplines and domains evaluated and the results obtained, have motivated curricular reforms to adapt what is taught to children and young people to prepare them for a globalized world, with a strong presence of information and communication technology. The Chilean students’ results have impacted the system, especially by highlighting its weaknesses, related to little improvement over decades, differences in learning achieved by different groups of students, and performance below than expected in the most economically and culturally advantaged sectors. To accomplish these challenges, the system has changed its organization and developed diverse strategies. Data provided by ILSA studies have been used to promote policies and programs for the improvement and strengthening of the most vulnerable groups and a general approach that promotes gender equality in education, politics, and labor. ILSA studies have also been a reference for innovation in educational assessments, allowing the country to evaluate and explore innovative learning areas such as digital and financial competences.

## Overview of Chilean Education System

The Political Constitution (1980) consecrates and ensures the right to education, which allows the full development of the person at different stages of his/her life; it establishes the compulsory nature of primary and secondary education and the duty of the state to finance a free system to ensure access for the entire population. The Constitution also enshrines freedom of education, and the right to open, organize and maintain schools and the right of parents to choose the school for their children. The Constitution also explains the need for an organic constitutional law that establishes minimum requirements and objective norms for the educational system. Since 2009, the General Education Law (LGE)^1^ normalizes Chile’s educational system framework (Ley N° 20.370 2009). The LGE defines the goals of school education, regulates the rights and duties of the members of the education community, establishes minimum requirements for completion of each of the education levels, and institutes a process for the recognition of education providers (Biblioteca del Congreso Nacional de Chile 2016).

Organizationally, the Chilean educational system is governed by the Quality Assurance System (QAS),^2^ which is mandated to guarantee good quality education for all students in the country. From the beginning of QAS, in 2012, the educational system comprises four institutions with different duties.

The Ministry of Education is the central institution of the QAS. Its purpose is to implement educational policy by granting official recognition to educational institutions, defining regulations, providing funding, and creating and supporting educational resources, learning standards, and pedagogical training. Other institutions of the QAS are the Superintendence of Education, the National Council of Education, and the National Agency for Educational Quality (Ley N° 20.529 2011).

At a local level, educational institutions differ by administrative dependence and by educational tracks. According to their administrative status: public schools (43.9% of total) are managed by local governments (municipalities) or local education services,^3^ and funded by the state (Ley N° 21.040 2017); private subsidized schools (48.9%) are managed by private entities and funded by the state^4^ (Ley N° 20.845 2016); and paid private schools (7.2%) are managed by private entities and funded exclusively by families. By 2018, there were 12,021 schools in Chile, serving 3.58 million students.

Chile’s current school system consists of eight years of primary education (educación básica), a combination of primary and lower secondary education (Grades 1st to 8th),^5^ and four years of secondary school (educación media), which corresponds to upper secondary education (Grades 9th to 12th). Primary education starts when students are six years old. In total, there are 13 years of compulsory education from kindergarten^6^ to 12th grade (Ley N° 20.710 2013).

Schools may offer primary education (mainly small rural schools, offer education for Grades 1st to 4th or Grades 1st to 6th only), secondary school education (Grades 7th to 12th), or both (complete schooling). Schools providers of upper secondary education offer humanistic-scientific education, technical professional (vocational) education, or both (polyvalent). The differentiation between humanistic-scientific and technical professional education occurs in 11th grade, and different curricula for each track accompany it. A small group of schools offers specific artistic education. For students with special needs, temporary or permanent, there are economical, human, and technical resources, and specific knowledge and assistance available. Students with special needs can attend regular schools where facilities and methodologies are adapted for them,^7^ or they attend special schools, organized by type of disability.

### The National Agency for Educational Quality in QAS

In recent years, several efforts have been implemented in Chile to improve quality and equity in the education system. Among the concrete measures to support the improvement of quality, meet the Framework for Good Teaching (published in 2003), the Framework for Good Management (published in 2005), in 2011 was established the Quality Assurance System^8^ of Early childhood education, Basic and Secondary Education and its Inspection (QAS). Within this system, the National Agency for Educational Quality inherited from the former Unit of Curriculum and Evaluation of the Ministry of Education the responsibility to evaluate learning outcomes of students, through national tests (“SIMCE” is a national census assessment conducted every year in particular grades in specific subjects from 1988) and other Indicators of educational quality mostly related with socio-emotional aspects, with also the implementation of ILSA studies. This implementation includes all the procedures and processes for sampling, translation, and adaptation of instruments, test administration, database elaboration, and the publication of a first national results report.

### Analysis and Dissemination of ILSA Studies Data and Results

The main results produced by ILSA studies are widely disseminated in the country by the National Agency for Educational Quality. Firstly, the data are released in press conferences with media coverage, the same day that the international report is presented to the world by the institution conducting the study, immediately after that the embargo is finished. After that occurs, the publication of national results reports with specific analyses are carried out, and seminars and workshops oriented fundamentally to teachers, principals, education faculties, present and explain the results, and train on the assessment methods developed by these international projects.

The National Agency for Educational Quality maintains a website in which each of the ILSAs is presented, with their specific characteristics: the assessed domain, target population, the general project design, and a series of materials offered to schools, the community-academic, and the general public. Among these materials are released instruments, like assessment frameworks, questionnaires, and test items. The international results reports are also included, as well as the national reports and any thematic reports developed in the Agency.

However, the possibility to conduct in-depth research with data from the ILSA studies is quite limited for the National Agency for Educational Quality. For this reason, it is widely promoted the development of secondary studies by researchers and academics. The ILSA studies’ databases with the manuals related to their management are made available to researchers and the community-academic. Agency organizes practical workshops where experts from the team train participants on statistical analysis that can be carried out. The new approaches and discoveries are fundamentals to enable the results to be used for public policy.

This emphasis on technical support and the required training was initiated with great vigor in the second PISA cycle where Chile participated (2006), to develop skills and make this technical knowledge available to research centers in the country. In fact, for the results of the PISA 2006, researchers from different centers were summoned, coordinated, and supported by a technical secretariat established in the Curriculum and Evaluation Unit of the Ministry of Education. With the contribution of an editorial committee composed of national experts, a first volume containing 11 articles was published. It was a selection that presented in-depth analyses and principal findings and lessons for educational policy from PISA 2006 (Ministerio de Educación de Chile 2009).

In 2012, the call was made directly by the Center for Studies of the Ministry of Education, within the frame of the “FONIDE DATOS PISA 2009 Extraordinary call”, to encourage the use of the large amount of information provided by the study and development of research aimed at various topics of which PISA delivers data. In this call, the Fund for Research and Development in Education (FONIDE) funded nine projects, each of which focused its questions on different educational process areas using PISA data (Centro de Estudios 2012).

After 2012, several workshops of database analyses have been carried out. The manuals with instructions together with all the materials and information available from ILSA studies is made available for educational communities, researchers, and policymakers for their use in the design of studies, projects, strategies, and initiatives that contribute to the improvement of the quality of the education that Chilean children and youth are receiving.

## Impact of ILSA on Chilean’s Educational Policies

Since the 70s, Chile has built a long history of participation in ILSA studies, covering various subjects and grades. Mostly led by the International Association for the Evaluation of Educational Achievement (IEA), the Organization for Economic Cooperation and Development (OECD), and the United Nations Educational, Scientific and Cultural Organization (UNESCO), these studies have been providing information to the Chilean education system related to mathematics, natural sciences, reading literacy, financial literacy, civic education, writing, computer literacy, among others. Chile’s systematic participation’s fundamental purpose is to acquire knowledge and international perspective, otherwise not available, to better guide systems, institutions, and practices, deemed of strategic importance to the country’s developmental goals (Cox and Meckes 2016).

Participation in ILSA studies has allowed the country to have relevant information to monitor the education system, the current curricula, public policies in education, and the programs that have been implemented, and incorporate international standards into national assessments and study frameworks. This participation also allows the country to compare Chile’s results regarding other participating countries that consistently obtain good results (Agencia de Calidad de la Educación 2019a).

The educational national evaluation guidelines are governed by the current National and international assessment Plan that allows projecting medium and long-term efforts to review and design educational policies. The plan reflects national agreements on how and what to evaluate, and the possibility of complement national and international evaluations regarding evaluation frameworks, educational context, subjects of assessment, educational policies, school management, and pedagogical practices. (Decreto N° 183 2016). The current plan determines that Chile is part of the development of Programme for International Student Assessment (PISA),^9^ Trends in International Mathematics and Science Study (TIMSS), Progress in International Reading Literacy Study (PIRLS), International Computer and Information Literacy Study (ICILS), International Civic and Citizenship Education Study (ICCS),^10^ and Regional Comparative and Explanatory Study (ERCE).^11^

For the year 2021, a new National and international assessment Plan will approve national and international evaluation guidelines for years 2021–2025.

Over the past 20 years, ILSA studies have contributed to Chile’s education policy by delivering information for decision-making at different levels. Mainly at: (a) Curricular adjustments and reforms designed by experts, (b) Educational system and its regulations, and (c) National assessment system.

### Curricular Adjustments and Reforms Designed by Experts

In 1988, before Chilean participation in modern international studies,^12^ the Unit of Curriculum and Evaluation of the Ministry of Education started to collect information about the level of students’ knowledge through the national standardized assessment named SIMCE. This assessment measures achievement of fundamental curricular objectives and minimum compulsory contents in Language, Mathematics, Natural Science and Social Sciences. However, through this national assessment, it was not possible to contextualize students’ learning regarding students’ achievements in other countries or to analyze the national curriculum, teacher training, or pedagogical activities regarding other educational systems.

The participation of Chile in ILSA studies revealed the challenges faced by the national school system regarding the improvement of student learning in different subjects. Despite observing good results in comparison to other Latin American countries, the distance to the average performance of all countries participating in these studies is considerable. These results, along with the content and cognitive domains of different subjects, have been used to support and nourish the curricular revisions and reforms.

The curricular adjustment of 2009 was the first extensive review and update of the curriculum for primary and secondary education since the late’90s.^13^ Among other documentation and inputs (social demands started by secondary students, curriculum analysis, studies of relevance, surveys, revision of other countries’ curricula, public consultations), the ILSA studies available up to that time were considered. This adjustment explicitly incorporated the information, both results and frameworks, obtained in TIMSS for Mathematics and Science, PISA for Language and Communication, and ICCS for Citizen training (Cox and Meckes 2016).

Learning objectives that were not part of the curricula then were identified and integrated. For example, “Earth sciences” was added to the contents of primary and secondary education in Natural Science, and civic contents related to formal political participation and relationships with the political system were added to the History and Social Sciences curricula. (Cox and Meckes 2016).

The 2009 National adjusted Curricula were understood as a curricular framework and from other instruments in which it was possible to address them. These instruments, with different purposes, were oriented to the achievement of the learning defined by the curricular frameworks.^14^

The analysis of international evidence related to higher achiever countries, weakness in Chilean students’ training, and topics emphasized in the international frameworks had allowed national curricula developers to establish requirements and sequence of the learning objectives for the subjects covered by these studies. Consequetly, the Learning Progress Maps were developed in 2007. They described the sequence in which a given competence, within the different curricular sectors, is typically developed throughout the school career (12 years), based on the learning opportunities prescribed in the curricular frameworks. Their purpose was to support teachers in the process of observing, analyzing, and monitoring the learning of their students (Ministerio de Educación de Chile 2007). Learning Progress Maps were replaced by Progression Of Learning Objectives, which have similar purposes and were also developed for each curricular sector, per grades.^15^

Performance levels of achievement were incorporated from the experience gain in international studies participation. These performance levels detail descriptions of what students know and are able to perform related to their performance in the national assessment SIMCE. From this information, qualitative information about students’ performance is delivered to schools to allow them to identify weaknesses of their students’ learning. Learning Progress Maps explained above and performance levels were developed using the reference frameworks from TIMSS, CIVED and PISA studies applied between 1998 and 2009.

From 2012 to 2019, new processes of curricular updates were developed affecting primary, secondary, vocational, and early childhood education. Especially for primary and secondary education, ILSA studies results were explicitly recognized and documented as an important source. An example of this is the mention made in the curriculum modification decree for 7th to 10th grades about learning outcomes and assessment frameworks, mentioning that this data allows matching the requirements of the national curricula with international requirements in different subjects (Decreto N° 614 2014).

In the case of Curricular Bases for grades 1–6, the revision of International assessments of learning applied in Chile (TIMSS, PISA, PIRLS, ICCS) and their assessment frameworks have allowed having comparative information to make decisions about the topics to be covered in each course, and the sequences of content and skills. (Ministerio de Educación de Chile 2018).

In the case of Curricular Bases for grades 7–10, the ILSA studies are widely mentioned, but not related to specific subject topics. Instead, it is indicated in a general way whether the framework or the study’s results (report) of a particular cycle were used to develop and revise the subject. For instance, in Language and Literature, they mentioned the PISA 2009 assessment framework and a document with Reading task samples published in Chile (Ministerio de Educación de Chile 2011). In the case of Mathematics, PISA 2003 and 2012 assessment framework, together with PISA 2009 and TIMSS 2011 national report are mentioned as sources. In the case of Natural Science, assessment framework 2009 and TIMSS 2011, together with PISA 2006 international report, were mentioned as sources (Ministerio de Educación de Chile 2015a).

### The Educational System and Its Regulations

Information from Chile’s participation in international studies has been broadly used as input for evidence-based decisions to revise, propose, and adjust educational policies and practices to improve the school system. Data collected through these studies have been used as a reference in law discussions and adjustments regarding the school system’s organization and financing.

Besides, international studies have been extensively used during discussion and design of laws dealing specifically with subjects assessed by some of these studies. For example, the Citizen Education Law^16^ was inspired by the poor results about Chilean students’ civic knowledge, as shown by the ICCS 2009 study. With this base, on May 15, 2015, the President argued for the need for legislation to mandate every school in the country to define and implement a plan for citizenship formation (Cox and Meckes 2016), and during that year, the chamber of deputies submitted a bill of law to establish the obligation of every school to have a citizenship education plan. ICCS 2009 conclusions were presented as background and proof of this subjetc’s lack of presence during school education (Biblioteca del Congreso Nacional de Chile (2018). Although citizenship education was already part of the primary and secondary education curricula as a transversal learning objective, the need to strengthen this area was highlighted. Several professionals with knowledge in the subject used ICCS data through the process of generation of the law that was approved in 2016 (Ley N° 20.911 2016). This law established the obligation for schools to define and implement the plan for citizenship. It stated the obligation for the Ministry of Education to promote the incorporation of a compulsory subject of Citizenship Education for the 3rd and 4th grades of secondary education. This new subject of Citizenship education for these grades was approved by the National Council of Education in February 2018 and begun to run in March 2019.

The decision to incorporate Financial Education as an explicit subject into secondary education’s national curricula was based on the results of financial literacy from PISA 2015. During law discussions, members of the National Congress, Ministers of Education, and other experts use international studies data to reinforce their arguments. The most common use of these references is to account for the school system challenges in light of the results obtained compared to other school systems. For example, based on international and national PISA 2015 reports, the Commission of Education of the Chamber of Deputies prepared a detailed document with PISA 2015 Financial Literacy results of Chilean 15-year-old students, for the legislative deliberation, and its particular requirements and deadlines (Biblioteca del Congreso Nacional de Chile 2017). The law was promulgated in 2018^17^ (Ley N° 21.092 2018). Among others, the relevance that the OECD gives to financial education in PISA was one of the main arguments for this modification. The PISA assessment framework was also considered to define the main learning objectives to be highlighted in the national curricula.

Beyond Chile’s need to improve learning outcomes, ILSA studies highlight school system inequities, allowing for discussions regarding the need for system reforms focused on vulnerable groups.

On the one hand, the international comparison has shown that performance differences by socioeconomic and cultural levels exist in all participating countries, but the degree of association between socioeconomic origin and school results varies considerably in different systems (Sandoval-Hernandez and Castejon 2014). From this, it follows that it is possible to develop a more equitable school system, an idea that was presented as a reference for the discussion of the educational inclusion law^18^ (Cox and Meckes 2016).

On the other hand, gender gaps have been brought to attention. Traditionally, female students in Chile obtained worse results in mathematics and science assessments, limiting their participation in STEM careers that are the ones with a better salary in the work market. Results from TIMSS and PISA showed that such a gender gap is not common to all countries, and of course, the difference cannot be attributed to innate ease for men in learning those subjects. To move towards quality and inclusive public education, in 2014, the Ministry of Education created the Gender Unit (UEG), a structure that is responsible for promoting the incorporation of a gender perspective in the Ministry's plans. The main goal is building a non-sexist education where everyone's capacities are recognized regardless of sex, identity, and gender. In that framework, the Ministry established in 2015 a plan for Gender Equality 2015–2018 that made a diagnosis using a series of educational and labor data, including international assessment studies. It proposed a series of measures to incorporate the issue of gender and the need to work in an integrated and synergistic way with different ministries (Ministerio de Educación de Chile 2015b). In February 2019, the Ministry of Education and the Ministry of Women and Gender Equity signed an agreement that includes concrete measures to continue and deepen the initiatives that seek to eliminate gender biases and stereotypes in classrooms and grant equal educational opportunities to women and men.

The opportunity given by these studies to obtain data from the international comparison allowed the country to understand that the inequities which could be considered structural or immovable until that time are not natural. Moreover, information about successful countries related to the management of these challenges encourages the observation of practices replicable in our school system. An example of this is the targeting of resources to vulnerable groups. Preferential grants are delivered to the schools where most vulnerable students attend to mitigate social inequalities and improve school experience. The comparative experience served as an inspiration for these educational policies, for example, the Preferential School Subsidy Law,^19^ and its extension and update (Villarroel 2019).

The results of reading in PISA for Chilean students in 2000 and the following years (2009 and 2012), national reading tests, among other inputs, have been used to show the urgency of improving Chilean’s reading skills and the need to face the problem with national policy. To date, two National Reading and Book policies have been defined and implemented in the country,^20^ together with plans to promote reading, composed by a series of initiatives for developing these habits since childhood. Currently, the National Reading Plan 2015–2020 is in place, supported by a large number of government and private entities, which seek to “promote the formation of a society of readers, in which reading is valued as an instrument that allows people to improve their educational level, develop their creativity, sensitivity and critical thinking” (Gobierno de Chile 2015).

### National Assessment System

Regarding the relationship between ILSA studies and Chile’s national assessment system, one of the most important goals has been validating through international comparison the national assessment system itself, its methods, approaches, and results, establishing coincidences between similar results, seeking explanations when trends have been different.

To improve the national assessment system, not only international evaluation frameworks have been used as a reference to learning objectives but also specific tools for updating and refine other aspects related to item development, sampling, and technical requirements for trend analysis, statistical methodologies, test score estimation, test formats, innovative subjects, among others.

There are countless examples where international studies have been reviewed as a reference to check, revise, or innovate different aspects of the national assessment system. To mention some of them:

(I) Psychometric technical aspects. From the Classical Test Theory to Item Response Theory (IRT). Since its origin in 1988, SIMCE, the national assessment system, used classical test theory as the main measurement model for data analysis. However, since 1998 a transition process into IRT started, having as principal reference the international studies test scoring experience. (Agencia de Calidad de la Educación 2012). All cognitive tests are currently calibrated and scaled using IRT methodology and progress is also being made in using these models for scoring the context questionnaires’ items.
(II) Inspired by innovative assessments carried out by the international studies, some domains have been raised as initiatives of national interest:
  PISA Financial literacy led to the generation of a national interdisciplinary workgroup composed of financial, education, and public policy institutions to think of a national financial education plan. Likewise, within the process initiated with integrating into the national curricula of financial education, some materials, courses, and training were developed by governmental institutions. PISA financial literacy study is used in most initiatives as reference.
  International Computer and Information Literacy Study (ICILS) and International Civic and Citizenship Education Study (ICCS) led to national assessment initiatives^21^ developed to collect national information based on the international studies’ frameworks and procedures.
(III) The development of the Quality and Context of Education Questionnaires related to the national assessment tests has been influenced by the student, parent, teacher, and principal questionnaires from different international studies. Inspiring has been the used question formats, contents addressed, and the analysis methodologies that are constantly quoted as a reference.
(IV) ILSA studies also have produced in the national assessment system a valuable knowledge in the technical teams regarding items construction, the inclusion of open-ended response items, and the development of coding guides and coding procedures (Cariola et al. 2011).

## Students’ Movement, a Fundamental Actor in the Generation of Changes in the Chilean Educational System

This section is a general synthesis of the social movements in which Chilean secondary school students have been involved since 2006. These movements have been autonomous, that is, they were not summoned by political parties, and we dare to say that they have given rise and have accompanied the entire reforms process and attempts to improve the Chilean educational system. Contextual elements are offered to the readers to to show how a large majority of students have developed their school careers in the last 14 years in Chile. On the one hand, authorities in the country discussed, redefined, and implemented policies related to various aspects of the educational reality. On the other hand, students actively participated or were spectators on the front line in massive movements in which they left classes for long periods to march, occupy the schools, and hold discussion forums, workshops, and other training and development instances. The process of making up the missing classes was usually very demanding. Students had to face extensions of the class calendar and condensation of tests and exams to achieve certification and promotion. Some schools, besides, participated in international assessments administered in the period. Although it is possible to say that there have been students’ activities and demonstrations every year, two of them are the most relevant because of their high call and broad participation or in the concrete effects they produced, they are the first, in 2006 and the longest, in 2011. We also include 2012, 2015 and 2018 to complete the picture, and because PISA study or other ILSAs were administered then.

These movements bring together students who attend mostly municipal and some subsidized schools, starting in Santiago, the capital city, but extending later to the regions in the North and South. The students who attend private schools and represent almost 8% of enrollment in the educational system have little or no participation. Among the most active participants, there is a group of schools called "emblematic". They are public schools, free of charge, oriented to academic excellence, with long tradition and prestige (Rivera and Guevara 2017). Traditionally they have been highly selective, but selection practices must be erradicated after the Inclusion Law is implemented. Emblematic schools were targeted at men or women separately, but this feature is slowly changing. They are among the best municipal lyceums in the country, and although in the last years their achievements have decreased, their students get very good results in the SIMCE tests and selection tests for the universities. They promote civic and republican values, and their students are usually active participants in the movements.

Even if not all the students or schools have actively participated, social movements have undoubtedly affected the way in which the daily activities in the schools were developed in the country and has implied modifications in relation to the teaching-learning processes, the school climate, the relationship between students and teachers, and school authorities. This situation has also generated changes in the attitudes and public behaviors of students as well as the perception that general citizens have of them. Interesting studies on these topics have been developed, but they are not presented here.

At the end of *April 2006*, the first uprising of secondary students (Revolución Pingüina) took place. It was the first national and massive social mobilization since the recovery of democracy (Garcia-Huidobro 2009). A center-left wing coalition led the national government. The students started to require local and specific demands (school pass for public transport and the elimination of the payment for the university selection test), but soon more in depth and more cross-cutting themes appeared, such as the defense of the right to education, the improvement of public education quality, the end of municipalization, a rejection of the privatization of education. The students’ objective was to repeal the Constitutional Law of Education (LOCE), the legal foundation of the educational system enacted by the Pinochet regime in 1990 (Bellei and Cabalin 2013), March 10, one day before the new democratically elected government assumed power. The movement included the paralyzation of activities in schools, massive street demonstrations, national school strikes, school occupations, and a strong presence in the press, getting important support from diverse actors and sectors of the society. The movement, which was later joined by university students, was active between April and June and then resumed in September and October, around five months of the school year’s ten months. The PISA 2006 test was administered between August 21 and September 7, and SERCE was administered between October 16 and 20.

As a consequence of this movement, the government convened a Presidential Advisory Commission for Education. With more than 80 members, this commission met throughout the second half of 2006 to propose to President in December a report that included a series of proposals to improve the education quality. Among several others, the first proposal was to replace the current LOCE with a new law that could give legitimacy to the educational institution and guarantees the right to good quality education (Torres 2003). The General Education Law N°20,370 was promulgated on August 17, 2009.

After minor episodes of demonstrations and other events in the country in the intermediate years,^22^ the students’ mobilizations emerged strongly again in *April 2011* and continued throughout the year. A coalition of right-wing parties ledthe national government. Started this time by university students, the movement questioned the root of the education market’s general model that has produced enormous inequalities among the population and significant indebtedness because the persons who access university training have to apply for a loan to finance it. Secondary school students also joined the movement with their specific demands. However, the essential general demands were: “No profit”, the obligation of the state to guarantee a free and good quality public education in secondary and university level, and the end of public education administered by the municipalities (Muñoz and Durán 2019). The first demonstrations began on May 12, and in December, many students were still mobilized, some of whom had to repeat the school year. As in 2006, the movement consisted of massive marches, strikes, occupation of school buildings, very massive street demonstrations, a strong presence in the media, and it also included secondary students’ hunger strikes (Arrue 2012). The movement managed to install the most important ideas raised by the students in the whole society, especially the critique towards a profit-based education system. It garnered much support from citizens, starting with parents, the general public, even among university authorities. National paralyzations called by unions with national representation, including teachers, to support the students occurred, and other groups with specif demands appeared in public life.^23^ It is necessary to point out that there was much repression towards the students. To reject the harsh repression of the Carabineros to dissolve a student march in Santiago downtown, the Students’ Federation of the University of Chile called on the public to show solidarity with the protesters by hitting the saucepans, which had been very popular more than 25 years before. The noise made by hitting pans and pots to support the students happened on August 4 night. This demonstration spread through various sectors, from the most popular and the middle classes, transforming itself into a new form of protest that accompanied the movement until its last massive activities and marches (Núñez 2012).

During the seven months of conflict, there were many attempts at dialogue between leader students and the government, with several government proposals, which were not accepted by the students. After three changes of ministers of Education, the year finished without clear solutions^24^ (Taller de Análisis de Contexto—Varios autores 2012), but the students had become more than protesters in the streets: they became political actors and relevant players in the educational policy debate (Bellei and Cabalin 2013).

In *2012*, the students’ movement continued with marches in April and June and several strikes and occupations of school buildings in the middle of the year. PISA 2012 test was administered between August 20 and September 12.

In *2015*, with a center-left coalition in the national government, a new educational reform was proposed, and its implementation started. The students’ movement reorganized, and there were protests between April and June. The teachers’ union called to an indefinite strike, which was extended from June 1 to July 27, in protest of the Teaching Career project proposed by the government. It implied that a big amount of students this year missed two months of classes. PISA 2015 test administration had to be delayed in Chile for that reason and was finally administrated between September 21 and October 10. In 2015, the National Agency for Educational Quality administered two other international assessment projects in Chile: PIRLS 2016, and ICCS 2016, both between October and November.

In *2018*, between May and July, mostly university but also secondary students held a feminist student mobilization. The movement sought to denounce and punish cases of sexual harassment and abuse by teachers and demanded a process of social change to eradicate the prevailing machismo and the structural patriarchal system. The movement’s demands include taking action against academics accused of sexual abuse, eliminating sexism from education, making changes to curricula, and training gender equality. The movement implied strikes, cultural activities, and occupation of schools and university buildings. The longest occupation lasted 74 days in the Law Faculty of Universidad de Chile, until July 9. PISA 2018 test was administered between August 20 and September 7. In 2018, the National Agency for Educational Quality administered two other international assessment projects in Chile: ICILS 2018 in September, and TIMSS 2019, between October and November.

Students were once again the protagonists of a movement dveloped between *October 18, 2019, and February 2020* in Chile. It was started by secondary school students who protested the rise in the ticket price of Santiago public transportation system and began to evade payment. The general public quickly and widely supported this action, transforming the movement from one focused on students, to one that concerned a large part of the country’s population. The police force harshly repressed it. Trying to keep the country under control, the government decreed a state of emergency and curfew in some cities between October 19 and 28.

However, the movement spread in the form of pacific protests on the one hand, and violent demonstrations on the other, to most of the country's large cities. The main demands were related to the high cost of living, low pensions for retirees, social and economic inequity of Chilean society, criticism of the privatization of health services and housing, a general rejection of politicians and the armed forces. The need to have a new constitution was raised as a fundamental issue.

This extended movement was called “Chile woke up” and consisted of a series of activities of massive street demonstrations in strategical places in the cities, students and workers strikes and paralyzations, barricades, and traffic cuts on the streets, protests from the houses making noise by hitting pans and pots at certain times. Some groups’ actions were also directed to destroy Santiago metro stations, public and private buildings, and looting of commercial establishments. National economic and social issues were the subject of conversation at all levels. Many people who had had a very passive attitude concerning their working life, and social, cultural, and political aspects began much more active participation.

There was a high degree of repression against the protesters that motivated that several international humanitarian entities sent observers and published reports about the high amount of human rights violations by agents of the Chilean state. The students’ vacations and summer season slowed down the movement, but an increase was expected in March with the return to schools. However, the COVID-19 pandemic paralyzed the actions.

## Main Marks of Educational Evolution Related to PISA

Chile has been participating in PISA since 2000, being part of every cycle so far, except for PISA 2003. Since PISA 2012, the country has also participated in innovative domains such as Financial Literacy, Global Competence, Problem Solving, Collaborative Problem Solving, and the Well Being questionnaire.

In Chile, PISA has raised questions covering both the performance on the main domains over time, and recognizing specific—not always positive—characteristics of the national school system. The country has been able to comprehend that although the Chilean students’ achievement is at the top within Latin America, it remains below the OECD mean performance in every assessed PISA domain.

**Fig. 1:**
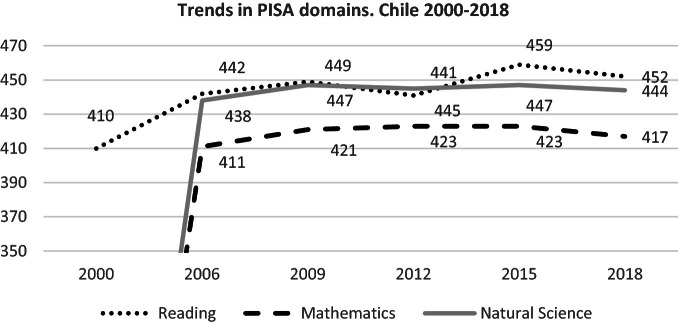
Trends in PISA domains. Chile 2000–2018. *Source* PISA 2018 Results Volume I. Table I.B1.10 [3/4]; Table I.B1.11 [3/4] Table I.B1.12 [3/4]

15-year-old students of Chile have the best relative performance in reading, science, and mathematics among the countries of the Latin American region that participate in the project. Additionally, trend analyses of Chilean PISA results have shown some statistically significant improvement in reading literacy, which tends to flatten out in the last cycles, while performance in mathematics and science has remained stable (see Fig. 1).

For Chile, and for most participating countries, there were no statistically significant changes in students’ performance in 2018. With an average performance of 487 in reading, most of the OECD countries outperform Chile, which obtained an average of 452 in reading. As far as OECD members are concerned, Chile obtained the third-lowest performance in PISA 2018 in reading, mathematics, and science, only surpassing Mexico and Colombia (OECD 2019).

**Fig. 2:**
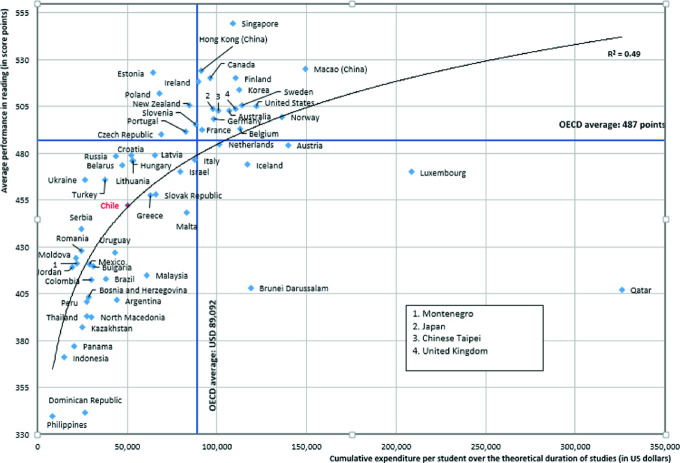
Cumulative expenditure per student during studies (in US dollars). *Source* OECD, PISA 2018 Database, Tables I.B1.4 and B3.1.4

Some notable differences may be observed comparing Chile and other countries with a similar accumulated expenditure per student between 6 and 15 years of age. On the one hand, Chile obtained reading results similar to Greece, Malta, and the Slovak Republic, although all of them have a higher accumulated expenditure per student. On the other hand, Ukraine, Turkey, and the Russian Federation have similar or lower accumulated expenditures per student but achieved better results than Chile (see Fig. 2).

The reading performance of students in Chile reaches the statistically expected value according to the education expenditure. On the contrary, for the other participating Latin American countries, students’ performance is below the expectation according to their spending.

Regarding the proficiency levels described by PISA, around one-third of students in Chile (31.7%) performed below Level 2 in reading at PISA 2018. PISA designates Level 2 as the base level of proficiency required to address reading-related issues demonstrating the capacity to use their reading skills to acquire knowledge and solve a wide range of problems. The proportion of students in Chile who obtained results below Level 2 was significantly higher than the OECD average of 22.7%. Chile also obtained a higher percentage than the OECD average of poor performance in science (35.3%). Most striking, over half of the 15-year-old Chilean youth obtained results below Level 2 in mathematics (51.9%).

PISA results show that Chile has difficulties in strengthening high-performance students who could help transform the country into a complex and knowledge-based economy later with their professional work. Only 2.6% of Chilean students obtained upper performance (levels 5 and 6) in reading, compared to the OECD average of 9.2%. Only 1.2% of students reached high mathematics performances compared to the OECD average of 9.4%, and only 1% achieved these levels compared to 7% OECD average in science (OECD 2019).

PISA results show that the Chilean education system reflects substantial inequities of Chilean society. 15-year-old students in Chile show a bigger variation than what is observed in OECD countries average in the Economic Social and Cultural Status Index (ESCS) distribution. Chile’s value is 1.03 and for the OECD average is 0.93, with 53 countries with less variation (more homogenous societies) and 23 with a greater variation in the index (more heterogeneous societies).

Although the effect of the socioeconomic and cultural status is very strong, Chile is not the country with the most significant effect of all. The strength of the relationship between ESCS and reading proficiency is expressed by the socioeconomic gradient, which refers to how well ESCS predicts the performance. In this indicator, Chile is quite close to the OECD countries average, with a value of 12.7 (OECD average is 12). There are 47 countries where the effect of ESCS is weaker than what it is observed in Chile, but also, there are 30 countries where the effect of ESCS is stronger than what it is observed in Chile. The countries with the strongest relationship between ESCS and reading performance show values between 18 and 21 and are Peru, Belarus, Hungary, Romania, and Philippines. On the contrary, the countries with the weakest relationship between ESCS and reading performance show values between 5 and 1.7 and are Montenegro, Hong Kong (China), Kosovo Republic, Baku (Azerbaijan), Kazakhstan, and Macao (China) (OECD 2019).

Specific results in Chile for main PISA domains are described below. Two important characteristics are identified within them, *Gender* gap and differences according to *Socioeconomic and cultural status*. Both will be addressed next.

### Reading

**Fig. 3:**
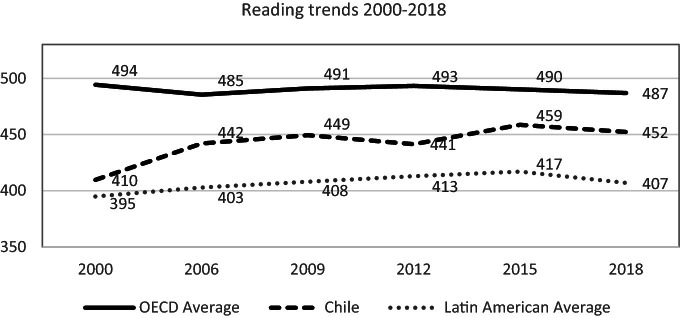
Mean reading performance, 2000 through 2018. *Source* Developed by Agencia de Calidad de la Educación with PISA 2000–2018 International Databases

Chilean students’ performance in reading has significantly improved since the first cycle, with 7.1 points of average change, per a 3-years period, between 2000 and 2018 (OECD 2019). However, the trend is less positive nowadays. Comparing results from most recent PISA cycles to PISA 2000, Chile stands out as one of the best countries in the Latin American region even though, it remains below the OECD average (see Fig. 3).

The following graph summarizes the trajectory of 15-year-old students in Chile for almost two decades and shows that there have been improvements that, although they have not continued, have not been reversed either.

The percentage of students below Level 2 decreased significantly between 2000 and 2009 (from 48 to 31%), and after that this decline stopped. It is crucial to reactivate this decline because that implies advancing justice and integration into society since although these citizens can decode and read a text, their reading competence does not allow them to receive all the information they need to carry out entirely various tasks, to inform themselves, learn new things, or entertain themselves.

The percentage of students who barely reach the minimum skills to enter society successfully (Level 2) has remained constant in the period (with percentages ranging between 28 and 35%). However, there have been small changes in the higher levels, with an increase in the percentages of students reaching levels 3 and 4 and those who have developed more advanced reading skills.

**Fig. 4:**
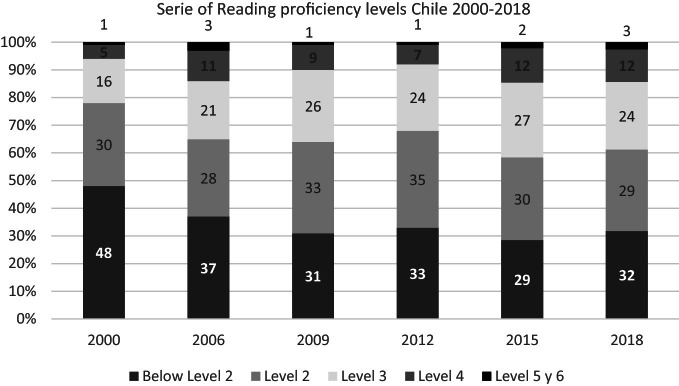
Reading proficiency levels , Chile 2000–2018. *Source* Developed by Agencia de Calidad de la Educación with PISA 2000–2018 International Databases

Despite the stable overall performance, the proportion of Chilean students performing at Level 5 or above (top performers) in reading is significantly higher in 2018 regarding 2009 and 2012, with 1.3% and 2.0% respectively (OECD 2019). This trend must also be deepened; it will imply advantages for individuals and the whole society (see Fig. 4).

It is possible to explain, at least partially, the improvement observed in 2006 due to the curricula updating implementation. Most students who took the PISA test in 2000^25^ were part of a population who had studied the primary education with the curricula defined and implemented by the military government, before the curricular reforms of 1996 and 1998. The reformed study programs were implemented gradually, starting in primary and then in secondary education. In fact, the students in 10th grade taking the PISA test in 2001 were the second generation that had studied that grade with the curricula established in 1998. On the contrary, students who took the test in 2006 were trained during all their educational careers with the curricula established in 1996 and 1998.

In the 2009 cycle, Chilean students showed an increase in the reading mean. Two reading tests (paper and digital) were administered in a group of countries at that cycle. Both tests were reported on the same scale and could be compared. In Chile’s case, students’ performances were significantly different, with a lower average in digital reading. Since PISA is a computer-based test since 2015, it could be expected that the reading mean in 2018 with a more robust scale because it is the main domain was lower or similar than the paper and pencil test and shows a smaller increase over time.^26^

#### Gender Gap

**Fig. 5:**
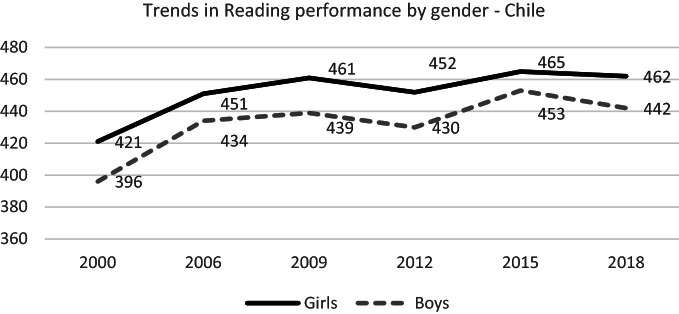
Mean reading performance, 2000 through 2018 by gender. *Source* Developed by Agencia de Calidad de la Educación with PISA 2000–2018 International Database

As in all participating countries, in Chile, girls show higher reading competences than boys do. This trend is consistently maintained over time (see Fig. 3). But it is interesting to notice that Chile is among the eight countries with the narrowest gender gap in reading (less than 20 score points): Argentina, Colombia, Costa Rica, Mexico, Panama, and Peru; all of them are Latin American countries with low average and B-S-J-Z (China) with the highest average in the cycle (OECD 2019) (see Fig. 5).

#### Socioeconomic and Cultural Status Differences

In PISA 2018, there were no notable changes in reading competence in any socioeconomic and cultural quintile (PISA ESCS Index, divided into five groups) for Chilean 15-year-old students regarding 2015. Historically the country has shown relevant inequities in the educational achievements between different social, economic, and cultural origins. The gap between the most disadvantaged students’ performance (quintile 1) and the most advantaged students’ performance (quintile 5) has been constant.

**Fig. 6:**
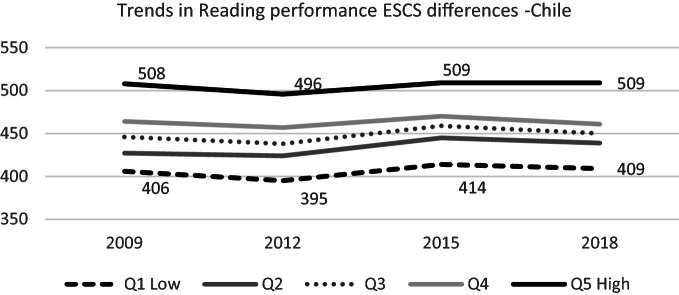
Mean reading performance, 2009 through 2018 by socio-economic and cultural quintile. *Source* Developed by Agencia de Calidad de la Educación with PISA 2009–2018 International Database

However, these advantaged students’ performance is not exceptionally high. It exceeds the current OECD average in the last PISA cycle, but is far from the countries’ average with the best achievements.^27^ On the contrary, it is possible to notice that quintile 2, the group with serious difficulties but maybe in the limit of extreme poverty, have improved consistently through these years (see Fig. 6).

On the national SIMCE reading test, it is possible to observe that the performance of the groups with the higher socioeconomic and cultural status in 10th grade^28^ has gotten worse without stopping since 2012 (Agencia de Calidad de la Educación 2019b, page 30).

**Fig. 7:**
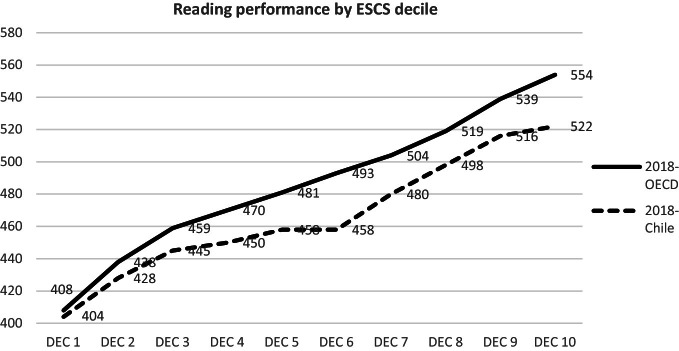
Mean reading performance by Decile of socioeconomic and cultural status. *Source* Developed by Agencia de Calidad de la Educación with PISA 2018 International Database

Similarly, it is remarkable that students currently belonging to the most socioeconomic and culturally disadvantaged group in Chile (ESCS decile 1) achieved similar performance to students of the OECD average who have similar conditions. On the contrary, students from Chile with higher socioeconomic and cultural resources (ESCS decile 10) perform significantly below their peers in the OECD (see Fig. 7).

This finding shows that although the Chilean educational system manages to produce small improvements in the most vulnerable sectors, has failed to improve the education quality, even in students with higher resources and more significant possibilities to develop their skills and achieve excellence levels.

This weakness was already identified at the beginning of the Chilean participation in PISA when it was clear that compared with students in the similar socioeconomic and cultural conditions in Latin America, Chile's elite did not stand out. “This means that, even though these young people are the ones who probably get the best results in national assessments, their schools and families should not be satisfied” (Ministerio de Educación de Chile 2004).

#### Reading Performance Explanatory Model

**Fig. 8:**
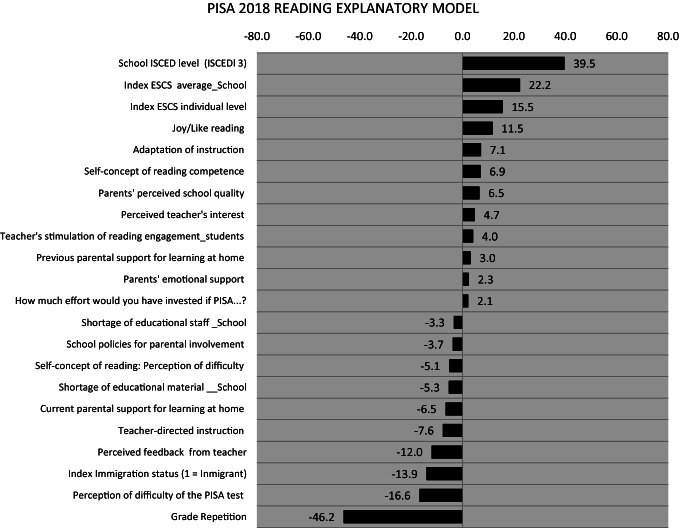
Reading Explanatory model. *Source* Developed by Agencia de Calidad de la Educación with PISA 2018 International Database. Note: Intercept Value: 446

The following exercise presents a multilevel analysis that is seeking to establish the relationship between a series of explanatory variables at the individual and school level and the average of Reading in 2018. The coefficients indicate how much the Reading average changes when the explanatory variables’ value changes (see Fig. 8).

The most evident aspect that this graph presents is the importance of students go on their school career without being left behind: the most positive effect is produced by attending a secondary school (ISCED 3) when one is 15 years old, while the most negative effect comes from having repeated a grade.

At the school level, the average of the students’ socioeconomic and cultural status has the most substancial effect on their performance. The families’ socioeconomic and cultural characteristics are not modifiable by the school. However, it is possible to generate measures that reverse the current social segregation in the Chilean school system to tend towards greater integration of students from different backgrounds within the schools.

At the individual level, enjoying reading and having a positive self-image as a reader are shown to have positive effects on achievement. Instead, feeling that reading is difficult has a negative effect.

Good teaching practices, reported by students, about adapting the instruction to different students, stimulating their engagement with reading, and showing interest in them have a positive effect. On the contrary, teachers who are too directive in their teaching have a negative effect on their students’ performance.

In turn, the parents’ emotional support and their perception that their children's school provides quality education have positive effects on performance.

On the contrary, the lack of material and human resources in schools, reported by the principals, negatively affects students’ reading learning.

It is also observed that more feedback from teachers and strong support from parents at the age of 15 years have negative effects. This information is consistent because students with low achievements generally receive more attention from their parents and teachers.

Finally, the graph shows that being an immigrant in Chile at age 15 has a negative effect on reading performance. The educational system and school communities must integrate immigrant students to make them have the same learning opportunities as other students. They arrived to stay; they need to be prepared. It will mean gain for the country, its equity, integration, and population capacities development. 

### Mathematics

**Fig. 9:**
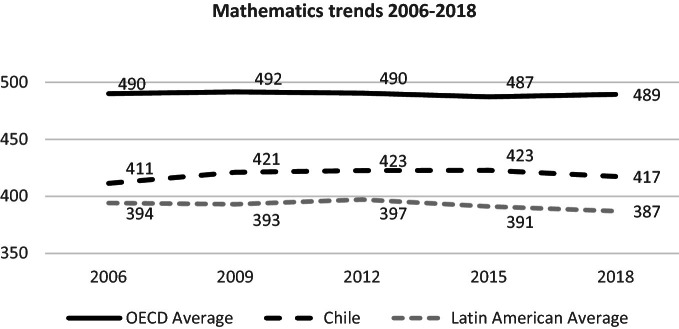
Mean mathematics performance, 2006 through 2018. *Source* Developed by Agencia de Calidad de la Educación with PISA 2006–2018 International Database

15-year-old students in Chile’s mathematics performance is significantly lower than the OECD average, although higher than the Latin American average. Through time, students in Chile obtain stable results and have not shown significant variations (see Fig. 9).

In all the PISA cycles, mathematics has proven to be the area in which Chile presents the most significant difficulty, with the highest percentage of students who don’t reach level 2, 51,9% (OECD 2019).

#### Gender Gap

Despite the stereotype that boys are better than girls in mathematics, boys significantly outperformed girls in mathematics in only 32 of the 79 countries and economies that participated in PISA 2018, and Chile is one of them (OECD 2019).

**Fig. 10:**
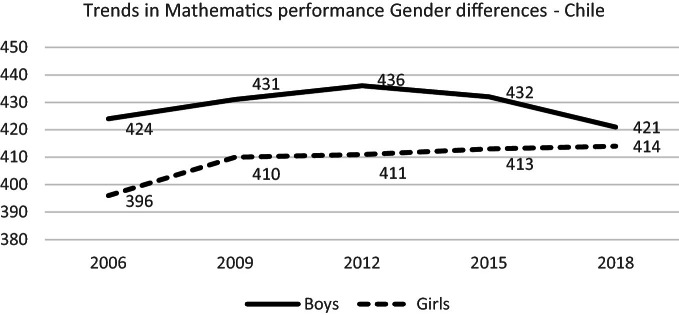
Mean mathematics performance, 2006 through 2018 by gender. *Source* Developed by Agencia de Calidad de la Educación with PISA 2006–2018 International Database

Systematically, girls in Chile show lower mathematics performance than boys. However, girls have shown a trend towards stability. On the contrary, boys’ scores behaved differently, and their performance fell significantly 11 points in 2018 compared to PISA 2015 (see Fig. 8). It is not clear, based on these PISA data, which can be the reason for this different behaviour. In any case, this information is consistent with the national test SIMCE where after some years of improvement for girls and boys of 10th grade, in 2014, boys showed a worsening in their performance meanwhile the girls showed stability. The situation has not changed lately (Agencia de Calidad de la Educación 2019b, page 35) (see Fig. 10).

It is not good news. Achieving gender equality in education is the first step to achieve a balanced society, but it is not a triumph that the difference is reduced because boys decline. It is essential that girls improve, but that boys also do.

### Socioeconomic and Cultural Status Differences

**Fig. 11:**
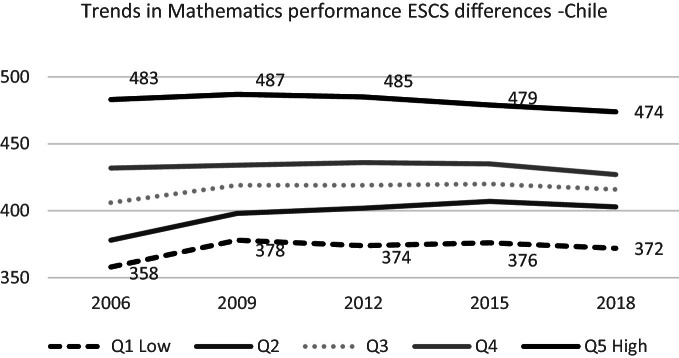
Mean mathematics performance, 2006 through 2018 by socioeconomic and cultural quintile. *Source* Developed by Agencia de Calidad de la Educación with PISA 2006–2018 International Database

None of the quintiles of different socio-economic and cultural status show significant changes in their math score between 2015 and 2018. However, the graph shows that quintile 2 has improved, and its trajectory of increasing is permanent (see Fig. 11). National SIMCE mathematic test results are consistent with this finding because, during the last decade, the gap between upper and lower groups has narrowed due to latters’ progress (Agencia de Calidad de la Educación 2019b, page 37).

### Natural Science

15-year-old students’ performance in natural science has not shown significant variations in the long term. Despite the stable overall performance, the proportion of Chilean students performing at Level 5 or above (top performers) in science shrank in 0.9% between 2006 and 2018 (OECD 2019, page 284). It is significant, even if it is a small percentage.

**Fig. 12:**
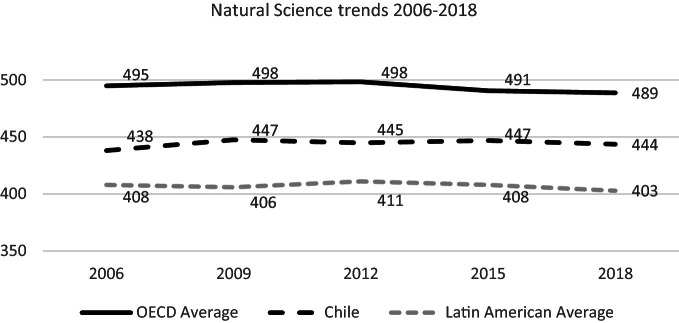
Mean natural science performance, 2006 through 2018. *Source* Developed by Agencia de Calidad de la Educación with PISA 2006–2018 International Database

Natural science results of students in Chilea are lower than the OECD average but higher than the Latin American average, and above the average of the participating countries in the region (see Fig. 12).

#### Gender Gap

In PISA 2009, girls managed to increase their scientific skills, but there have been no considerable changes since then. Boys have not shown changes through cycles.

**Fig. 13:**
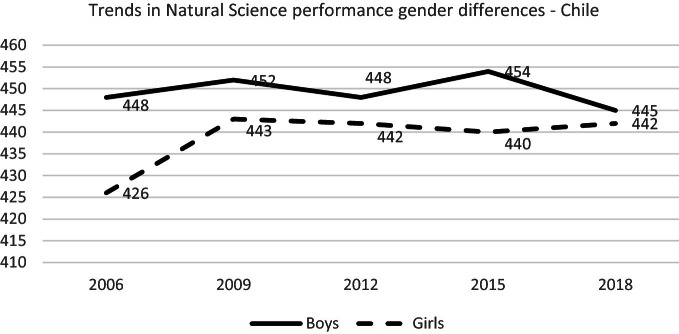
Mean natural science performance, 2006 through 2018 by gender. *Source* Developed by Agencia de Calidad de la Educación with PISA 2006–2018 International Database

PISA 2018 showed no significant differences in natural science by gender. Compared to previous cycles, more equity is observed, but this was due to a significant drop in boys’ results and non to a significant increase in girls’ performance (see Fig. 13), as also happened in mathematics. On the national SIMCE science test, it is possible to observe the same trend; boys of Grade 10 have reduced their score since 2014, and girls remain stable (Agencia de Calidad de la Educación 2019b, page 40).

#### Socioeconomic and Cultural Status Differences

**Fig. 14:**
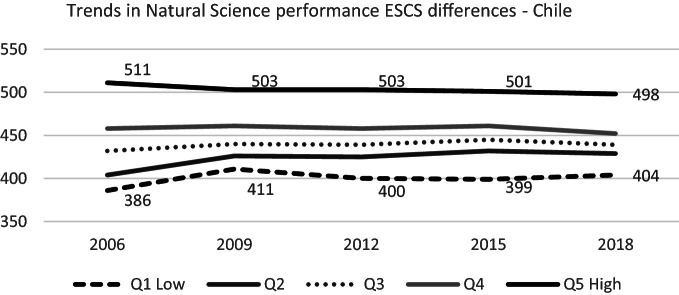
Mean natural science performance, 2006 through 2018 by socioeconomic and cultural quintile. *Source* Developed by Agencia de Calidad de la Educación with PISA 2006–2018 International Database

There are no significant scientific competence changes for any quintile of socioeconomic and cultural status in PISA 2018 regarding 2015. However, in the long term trend, it is possible to observe that while, the highest quintile is slowly reducing, the two lowest quintiles tend to increase their scores, especially the second, which shows a sustained improvement. This finding is consistent with what the national SIMCE science test shows: the most advantaged group has reduced their score since 2012; meanwhil,e the two lowest quintiles have remained stable (Agencia de Calidad de la Educación 2019b, page 41) (see Fig. 14).

## Conclusions and Recommendations

Since the beginning of the ‘90s, Chile is carrying out various educational reforms. From the end of that decade, data collected from international studies are fundamental pieces of the information available within the educational system to monitor its development and the achievement of its objectives.

For 20 years PISA has shown that the students’ performance in Chile has remained without much variation, especially in mathematics and natural sciences. In reading, improvements were observed between 2006 and 2009, but the situation has remained stable after that period. The few observed improvements correspond to the most socioeconomically and culturally disadvantaged students, which is very positive. It shows that the measures aimed at strengthening these groups and the schools that serve these students have made some progress.

Continuos curricular reforms have been made to adjust teaching to the changing demands of the times and the world globalization, but without a doubt, they are not enough to ensure that the majority learn and students who reach levels of excellence emerge.

The national context of recent years, related to social movements and educational adjustments, highlighted the need to make structural changes in the Chilean education system, both concerning the laws and regulations that govern its operation and its practices.

Objective data from the national evaluations periodically carried out by the SIMCE, related with no improvements in the students’ achievement and significant differences between groups within the country, made evident the lack of quality of the Chilean educational system. ILSA studies have made a substantive contribution evidencing the system’s weaknesses in the international comparison, highlighting high levels of gender and socioeconomic inequity, and the low quality of education received by large masses. Specifically, one of Chile’s main concerns is the significant proportion of students of secondary education underperforming in all PISA domains. It is vital to focus efforts to mobilize students at least to Level 2 in all domains, the minimum threshold to be able to join the society.

PISA and other international studies have also shown the Chilean education system’s inability to enhance the number of high-performing people who could help improve the country in different innovation areas. The country's efforts to improve low student performance include policies seeking to raise outcomes for those coming from less advantaged backgrounds, strengthening early childhood education, and early intervention mechanisms in case of difficulties. Policies should also include measures to promote excellence for all students and strengthen the students’ performance at higher proficiency levels (OECD 2018).

Data show that efforts to deliver more resources to schools to serve and retain in the system, especially the most socio-economically and culturally disadvantaged students, have the effect of making them learn more and be more competent, which is a goal of justice and integration into society. More efforts and efficiency are lacking, but it is going in the right direction.

International studies have been used extensively to improve national curricula by incorporating the knowledge and requirements that are internationally recognized as necessary to face present and future challenges, both concerning preparation for working life and citizen participation. ILSA studies will continue to be a reference in the national Education System. This is how, for example, the financial literacy evaluation framework will undoubtedly be used for the implementation of the law that integrates this subject to the curriculum of III and IV secondary grades. It is important to point out that based on international data analysis, some suggestions can be drawn. For example, the exercise presented with the reading scores (see Fig. 8) shows some aspects that, in each particular situation, can be considered and modified with concrete actions from the national policy, educational institutions, and families. 1.Although the practice of repetition has decreased, it is still used in the country. Evidence showing its ineffectiveness and even worse results provide arguments for seeking alternatives. Early detection and remedial interventions should be the solution to support students facing learning disabilities. Legislation, together with teaching practices and management in schools, should aim at this objective. 2.The country needs to advance in the equity of the educational system. It is fundamental that all schools, without distinction, may have the necessary, high-quality human and material resources to carry out their tasks. The educational system has to provide the means to make this possible. There are huge expectations that the non-selection, the reinforcement of public education, and the end of co-payment in schools that receive state funds will eventually produce a situation of a similar offer of quality education that families can access. This chain of facts will promote socio-economic integration that would reduce the segregation in schools, which replicates the society’s existing. QAS must develop its mission, and all the public institutions work coordinately to promote and facilitate that the dispositions are fulfilled, and the goals can be accomplished. 3.Due to the high diversity of students in the same classrooms, teachers need to develop the capacity to adapt their instruction to different students, which is related to show interest in all of them. For that reason, it is necessary to train the teachers effectively in methods and approaches that allow them to keep and manifest faith in all their students’ capacity. In that way, they will not consider useless to try different methods for students with difficulties. Teachers also need to be trained to stimulate the students, proposing them appealing challenges, and supporting their discoveries instead of being too directive in their teaching. 4.Given the enormous importance of the student’s ability to enjoy reading and have a healthy self-image about his/her capacity as a reader, it is clear that the first to develop this feature in children are the parents. They can encourage the children to read since the first years of life, reading for them or accompanying them while they read. Having parents who read recreationally also fosters a love of reading. 5. Depending on their particular reality, the schools may generate free and recreational reading spaces, using the available resources, both printed and digital, that allow students to venture into their motivations and interests and thus develop a taste for reading. 6.Specifically related to reading again, the educational system should generate training instances for practicing teachers in reading didactics and new methodologies to encourage reading in children and young people. Of course, schools need to count on physical and digital materials to promote reading for different purposes, starting from the youngest students.

We must recognize that despite decades of efforts, the Chilean education system, in general, remains deficient. Proof of this is that many of the expected achievements have not been accomplished even after the policies’ implementation, changes in the governments, and new reforms. Tensions persist in the system, as well as low students’ results in national and international studies, with stagnant indicators for years, and unmet aspirations for vast sectors.

Social movements with students as protagonists could be carrying out a long-term cultural and social transformation. They contribute to the generation of new citizens concerned with transforming society and its model, reflected in the design of its public policies in general. This aspect is very positive for the students themselves and the democratic system, but it is also true that it can difficult them to learn and develop the competencies that schools must provide, which is a high cost.

The current state of affairs, with an active, participatory, and demanding citizenry, partially—at least physically—stilled by the planetary emergency brought about the COVID-19, has meant time for reflection, study and the preparation of strategies to apply after the health emergency when it is necessary to face also the deepening economic and social crisis.

Education in Chile is indebted to the country. It should and can improve, and it is widely expected that citizens and authorities can agree peacefully and in democratic channels the best ways to get it. International studies will continue to monitor the teaching-learning processes. They will offer a comparison with similar and different educational systems, calling for reflection, searching for possible solutions and strategies for the identified problems. All these together will allow in the future—hopefully not too far away—education becomes a useful tool of personal development, satisfaction and social mobility. Then, the country will have more capacity to prepare all the people to decide their lives, reach their goals, become useful and committed citizens with their community to have a more equal, fair and balanced society.

## Footnotes

1 Law N° 20.370 (2009), General education law (LGE). It replaced the Constitutional Organic Law of Education (LOCE), Law N° 18.962 in force since March 10, 1990.

2 Ruled by Law N° 20.529 (2011), National system for the quality assurance of early childhood, primary and secondary education and its auditing.

3 Law N° 21.040 (2017), creates Public Education System (transfer schools from municipalities administration to local education services administration).

4 Law N° 20.845 (2016), School Inclusion law. Regulates students’ admission, eliminates shared financing, and prohibits profits in educational institutions receiving contributions from the state.

5 Law N° 20.370 (2009), changes basic and secondary education structure into six years of primary and six years of secondary education. Although it is approved, this measure has not been implemented yet.

6 Law N° 20.710 (2015), establishes the obligatory of the second transition level (kindergarten) and creates a free financing system from the middle level.

7 School Integration Program (PIE). Since 2009, this program seeks to educate children and youth with disabilities for part or all the time in regular schools. It includes providing of additional economic resources to the schools so that they can be used in the hiring of human resources, training of personnel, acquiring material resources, and in the generation of collaborative spaces to meet these students’ special educational needs.

8 Law N° 20.529 (2011). Creates the SAC, Quality Assurance System (QAS).

9 Developed by the Organisation for Economic Co-operation and Development (OECD).

10 All these studies are developed by the International Association for the Evaluation of Educational Achievement (IEA).

11 Developed by the United Nations Educational, Scientific and Cultural Organization (Unesco) through its Latin American assessment laboratory (LLECE).

12 Chile took part in the IEA’s Six Subject Survey in the late 60s.

13 New frameworks for primary and secondary education were promulgated in 1996 and 1998, respectively, and the study programs derived from them were gradually applied between 1997 and 2002. These frameworks were partially updated in 2002 and 2005.

14 Study plans, study programs, progress maps, SIMCE performance levels, and school texts.

15 Example for Language and Communication 1st to 6th grade of primary education. Progresión de objetivos de aprendizaje Lenguaje y comunicación. En: https://www.curriculumnacional.cl/614/articles-71255_archivo_01.pdf.

16 Law 20.911 (2016), creates a citizen education plan for educational institutions recognized by the state. It establishes that preschool, primary and secondary education must have a Citizen Training Plan.

17 Law N° 21.092 (2018). Modifies the LGE to include financial literacy contents in secondary education.

18 Law N° 20.845 (2016). School inclusion law. It regulates students’ admission, eliminates shared financing and prohibits profit in educational institutions that receive contributions from the state.

19 Law 20.248. Enacted in 2008, updated in 2011, 2016 and 2019.

20 Política Nacional del Libro y la Lectura 2006–2010 y Política Nacional de la Lectura y el Libro 2015–2020, both published by the National Council of Culture and Arts.

21 Link to Citizen national assessment https://www.agenciaeducacion.cl/evaluaciones/estudios-nacionales/. Link to Computational national assessment https://www.enlaces.cl/evaluacion-de-habilidades-tic/simce-tic/presentacion/.

22 On February 27, an earthquake and subsequent tsunami that struck central and southern Chile, with an intensity of 8.8 degrees on the Richter scale. The fatal victims reached a total of 525, nearly 500 thousand homes suffered serious damage and an estimated 2 million people were affected. It was the worst natural tragedy experienced in Chile since 1960.

23 Mobilizations of poor people without access to housing and environmentalists, as well as the struggle of the Mapuche people and sexual minorities, among others.

24 On January 24, 2018, the National Congress approved one of the most important educational reform of the late years, the free education in higher education, understood as a benefit to which persons apply individually and accessed by students who belong to the lowest income quintiles of the country and are registered in higher education institutions attached to this system.

25 Chile participated in a group of 11 countries that replicated the study in 2001. Their results were expressed on the same scale and included in the report with all the countries, in 2000 and 2001.

26 Cycle 2012, where the main domain was mathematics, showed a slightly strange behavior for the minor domains reading and science in Chile. For both areas, there was a decrease, even if it was not statistically significant.

27 Countries with the highest achievements in PISA 2018 Reading: BSJZ-China (555), Singapore (549), Macao-China (525), Hong Kong-China (524), Estonia (523), Finland (520), Canada (520).

28 10th grade is the national modal grade for 15-year-old students.
